# Identification of super enhancer-associated key genes for prognosis of germinal center B-cell type diffuse large B-cell lymphoma by integrated analysis

**DOI:** 10.1186/s12920-021-00916-z

**Published:** 2021-03-04

**Authors:** Xi Li, Yan Duan, Yuxia Hao

**Affiliations:** 1 Department of Lymphoma, Shanxi Bethune Hospital, Shanxi Academy of Medical Sciences, Taiyuan, Shanxi People’s Republic of China; 2 Department of Critical Care Medicine, Shanxi Provincial Cancer Hospital, Taiyuan, Shanxi People’s Republic of China; 3 Department of Gastroenterology, Shanxi Provincial People’s Hospital, 29 shuangtasi Rd, Taiyuan, 030012 People’s Republic of China

**Keywords:** Diffuse large B-cell lymphoma, Germinal center B-cell type, Super enhancers, Weighted gene co-expression network analysis, Overall survival

## Abstract

**Background:**

The pathogenesis of germinal center B-cell type diffuse large B-cell lymphoma (GCB-DLBCL) is not fully elucidated. This study aims to explore the regulation of super enhancers (SEs) on GCB-DLBCL by identifying specific SE-target gene.

**Methods:**

Weighted gene co-expression network analysis (WGCNA) was used to screen modules associated with GCB subtype. Functional analysis was performed by gene ontology (GO) and Kyoto Encyclopedia of Genes and Genomes (KEGG) enrichment. H3K27ac peaks were used to identify SEs. Overall survival analysis was performed using Kaplan–Meier curve with log-rank and Breslow test. The effect of ADNP, ANKRD28 and RTN4IP1 knockdown on Karpas 422 and SUDHL-4 cells proliferation was analyzed by CCK-8. Karpas 422 and SUDHL-4 cells were treated with bromodomain and extra-terminal domain (BET) inhibitor JQ1, and the expression of ADNP, ANKRD28 and RTN4IP1was measured by qRT-PCR.

**Results:**

A total of 26 modules were screened in DLBCL. Turquoise module was closely related to GCB-DLBCL, and its eigengenes were mainly related to autophagy. There were 971 SEs in Karpas 422 cell and 1088 SEs in SUDHL-4 cell. Function of the nearest genes of overall SEs were related to cancer. Six SE-related genes associated with GCB-DLBCL were identified as prognostic markers. Knockdown of ADNP, ANKRD28 and RTN4IP1 inhibited the proliferation of Karpas 422 and SUDHL-4 cells. JQ1 treatment suppressed ADNP, ANKRD28 and RTN4IP1 expression in Karpas 422 and SUDHL-4 cells.

**Conclusions:**

A total of 6 SE-related genes associated with GCB-DLBCL overall survival were identified in this study. These results will serve as a theoretical basis for further study of gene regulation and function of GCB-DLBCL.

## Background

Diffuse large B cell lymphoma (DLBCL) is a malignant tumor composed of large B lymphoid cells with a diffuse growth pattern. DLBCL has obvious heterogeneity and invasiveness, mainly divided into germinal center B-cell type (GCB), activated B-cell type (ABC) and unclassified type (UNC) [1, 2]. GCB-DLBCL is believed to originate from germinal center blasts and is characterized by high expression of BCL6 and high frequency mutations in immunoglobulin genes [3]. At present, one of the important challenges in GCB-DLBCL treatment is the low detection rate in the early stage, which limits the choice of clinical treatment method and leads to poor prognosis [4]. Genetic abnormalities and biological changes are important starting factors for the occurrence and evolution of GCB-DLBCL [5]. In recent years, the role of epigenetic changes in GCB-DLBCL progression has received increasing attention [6, 7]. Therefore, exploring new molecular markers is important for the early diagnosis and treatment of GCB-DLBCL.

Studies have shown that cancers are complex diseases with multiple factors and stages, and are regulated by multiple genes [8]. Systematic analysis of multiple genes is helpful to understand the mechanism of disease and provide a theoretical basis for improving diagnosis and treatment strategies [9]. Weighted gene co-expression network analysis (WGCNA) is a widely used systems biology method, which links complex clinical phenotypes and constructs corresponding networks based on the correlation between changes in gene expression signal values. Compared with differential expression analysis, WGCNA is more able to analyze the changes in the overall biological process, making it possible to identify multiple pathogenic genes and therapeutic targets at the same time [10].

Epigenetic changes at the early stage of DLBCL are reversible, making it possible to treat DLBCL at the epigenetic level [11, 12]. Enhancer is an important part of epigenetic regulation, which regulates gene expression by changing chromatin state through histone protease. Enhancers are *cis*-elements that promote gene transcription. After forming a complex with enhancer-binding protein, enhancer activates the transcription of related genes by interacting with transcription factors bound to the promoter [13]. Richard A. Young et al. first proposed the concept of super enhancer (SE) on the basis of genome-wide identification and functional characteristics of enhancers [14]. SE is a large cluster of transforming activity enhancers that drives the expression of cellular identity gene [15]. The expression of many key oncogenes is driven by SEs. Compare with normal cells, tumor cells construct SEs on oncogenes during tumorigenesis and recruit enhancer-binding proteins to drive gene expression [16]. Studies have shown that histone H3K27ac modification is the preferred marker for the identification of super enhancers [17]. Prediction of specific cell type SEs by histone H3K27ac modification can be used to explore key transduction factors specific to cell types.

In this study, WGCNA combined with SE molecular markers identification were used to determine GCB-DLBCL-specific key genes. The effect of key genes on GCB-DLBCL progression was verified at cellular level. The findings of this study are helpful to understand the molecular mechanism of the pathogenesis and progression of GCB-DLBCL.

## Methods

### WGCNA

Gene expression microarray data of DLBCL (GSE117556) including 255 ABC, 543 GCB and 130 UNC were obtained from Gene Expression Omnibus (GEO) (https://www.ncbi.nlm.nih.gov/geo/) database. The “WGCNA” package of R was applied for gene co-expression network construction. The soft threshold power was calculated based on pickSoftThreshold function. FlasClust was used to cluster genes and converted into an adjacency matrix, and then the topological overlap matrix (TOM) was transformed. Genes were clustered into different modules through the dynamic tree cut method with minimal size at 30. Modules with height value less than 0.2 were merged into the same module. Then, module eigengene (ME) was calculated. Pearson correlation analysis was used to assess the correlation between the MEs and DLBCL subtypes.

### Gene ontology (GO) analysis and the enrichment of metabolic pathways

GO annotation and Kyoto Encyclopedia of Genes and Genomes (KEGG) analysis were performed using “clusterProfiler” package with the cut-off values of enrichment > 2 and P value < 0.05.

### Screening of SEs

H3K27ac ChIP-seq data of Karpas 422 (ENCSR660IQS) and SUDHL-4 (GSE69558) cells were downloaded from Encyclopedia of DNA Elements (ENCODE, https://www.encodeproject.org/) and GEO databases, respectively. Short reads (50 bp, single end) were aligned to the human reference genome (hg19) using bowtie aligner version 2.2.452. Reads with multiple alignments were removed with samtools (v1.1) and de-duplicated with picard (v1.130). Processed bam files were subjected to identify peaks and calculate ChIP-seq tags using HOMER algorithm findPeaks tool with the parameter of finding histone-enriched regions (-style histone). Enhancers were defined as the regions where H3K27ac signals were enriched. Encryption regions within 12.5 kb between each other were stitched together to obtain enhancer clusters. Enhancer clusters were ranked based on H3K27ac signal values using HOMER algorithm super enhancer tool. The tangent points with tangent slope > 1 was identified as SEs, and points with tangent slope ≤ 1 were typical enhancers. Signal peaks of H3K27ac was visualized using UCSC Genome Browser.

### Survival analysis

The GCB-DLBCL patient population in the gene expression profile dataset with the accession number GSE117556 in GEO database was used to verify the overall survival. Kaplan–Meier survival analysis and log-rank test were used to compare the overall survival of patients. Benjamini and Hochberg method was used for multiple hypothesis testing. The hazard ratios (HRs) with 95% confidence interval (CI) of genes that significantly affect the prognosis were shown in forest plots.

### Cell culture, treatment and transfection

Karpas 422 and SUDHL-4 cells were purchased from American Type Culture Collection (ATCC, Manassas, VA, USA) and cultured in RPMI -1640 medium with 10% FBS (Gibco, Waltham, MA, USA), 1% penicillin and 1% streptomycin at 37 °C, 5% CO_2_. To explore the effect of JQ1 on the expression of ADNP, ANKRD28 and RTN4IP1 in Karpas 422 and SUDHL-4 cells, cells were cultured in RPMI-1640 medium containing 0, 1 or 2 μM JQ1 (CSNpharm, Shanghai, China, Cat # CSN13058) for 24 h.

For ADNP, ANKRD28 and RTN4IP1 knockdown, siADNP, siANKRD28, siRTN4IP1 and siNC were purchased from GenePharma (GenePharma, Shanghai, China). siADNP, siANKRD28, siRTN4IP1 and siNC were transfected into Karpas 422 and SUDHL-4 cells using Lipofectamine 2000 (Invitrogen, Gaithersburg, MD, USA) according to the manufacturer’s instructions.

### Cell proliferation assay

Cells were seeded into 96-well plates at 1 × 10^3^ cells/well and cultured for 24, 48, 72 h. Cell Counting Kit-8 (CCK-8) was used to assess the cell proliferation according to the protocol. The absorbance at 450 nm was detected using Thermo Fisher Multiskan FC (Thermo Fischer Scientific, Waltham, MA, USA).

### qRT-PCR

Total RNA of cells was extracted using Trizol (Thermo). cDNA was reverse transcribed using PrimeScript RT reagent Kit with gDNA Eraser (Takara, Dalian, China). qRT-PCR was performed using SYBR Green qPCR Master Mix Kit (Takara) according to the protocol. GAPDH was selected as the internal standard. The amplification condition was as following: 95 °C for 10 min, followed by 40 cycles each at 95 °C 15 s, 65 °C 30 s, 72 °C 30 s. The relative expression levels were calculated using 2^−∆∆Ct^ method. The primer sequences were shown in Additional file 1: Table S1.

### Statistical analysis

Statistical analysis was carried out using SPSS 22.0 software (SPSS standard version 22.0; Chicago, IL; USA). The differences among different groups were compared by one-way of variance (ANOVA). The differences between two groups were compared by Student’s *t*-test. *p* < 0.05 was considered as significant difference.

## Results

### Construction of the weighted gene co-expression modules of DLBCL

**Fig. 1:**
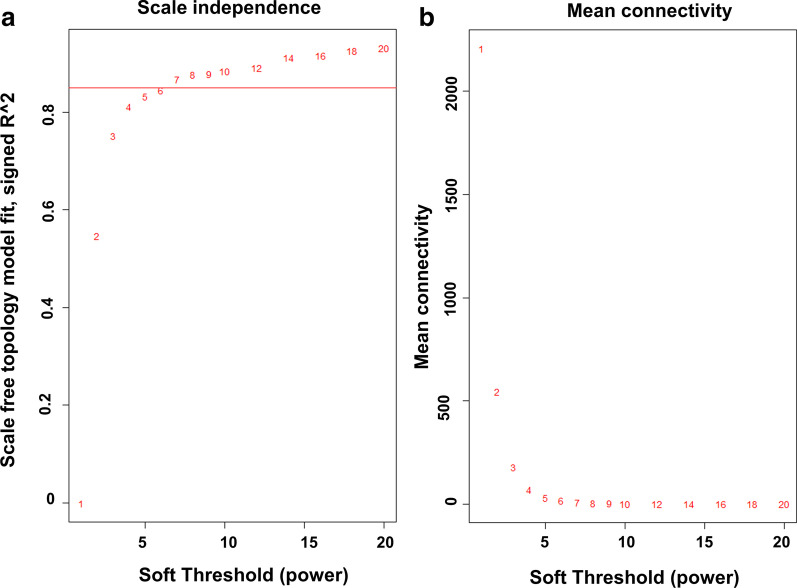
Estimation of the soft threshold power. **a** Scale independence analysis. **b** Mean connectivity analysis for soft threshold powers

In order to construct the co-expression network, we analyzed the gene expression microarray data GSE117556, including 255 ABC, 543 GCB and 130 UNC. The genes in this data set were generally well-expressed, and all patients had complete clinical data (diagnostic variables, treatment, treatment response, progress status, and follow-up time). A total of 20,297 genes were used for WGCNA analysis. Network topology analysis was applied to determine the appropriate soft threshold power. Normally, the scale-free topology module fit index (R^2^) is greater than 0.85, indicating that the network complies with the requirements of non-scale distribution. Therefore, the soft threshold power was set as *β* = 7 in this study (Fig. 1).

**Fig. 2:**
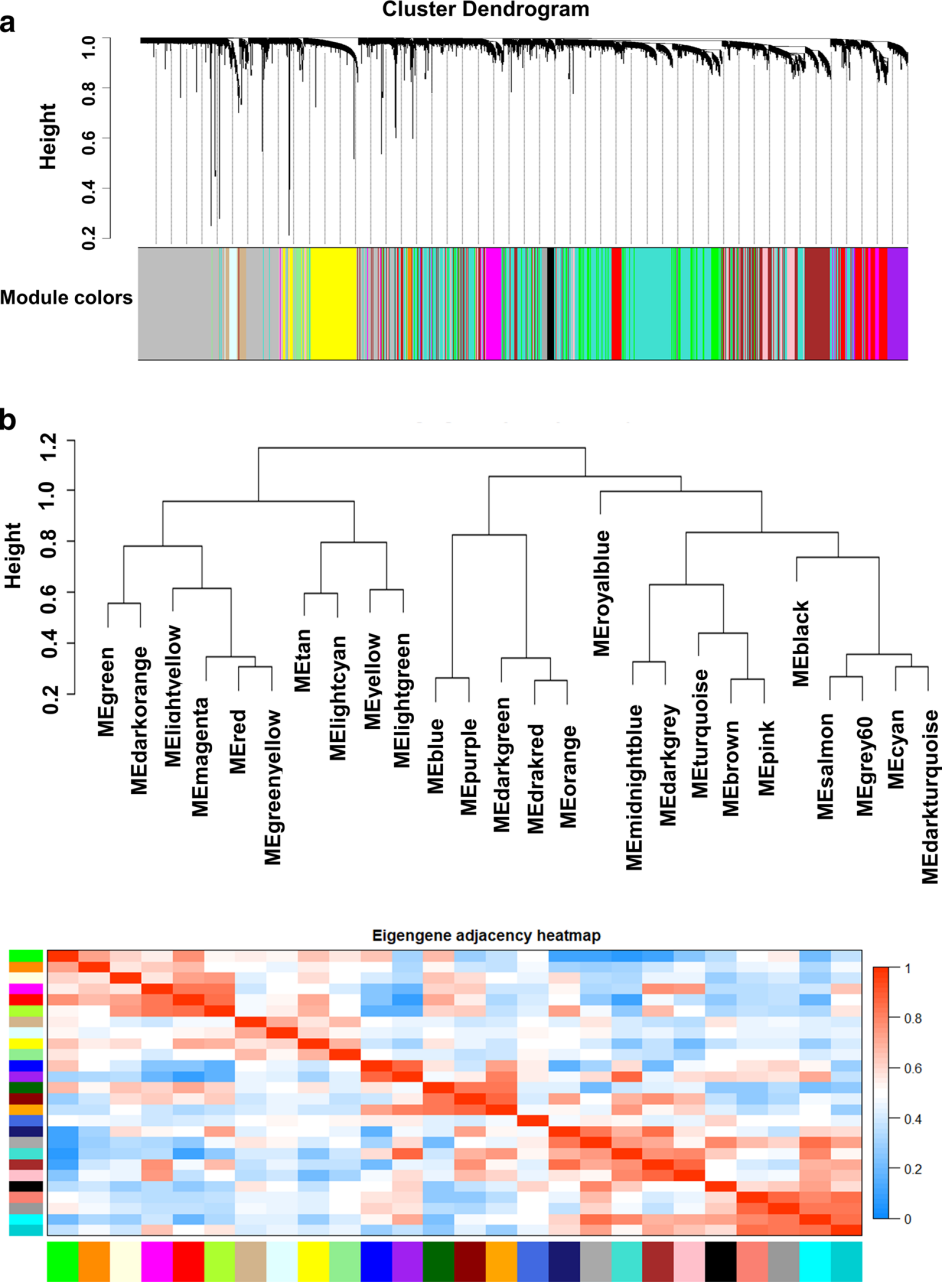
The weighted gene co-expression modules construction. **a** Cluster dendrogram of genes. **b** Hierarchical clustering dendrogram and eigengene adjacency heatmap of the 26 modules with the threshold as height value > 0.2

Subsequently, the genes were clustered according to the dissimilarity. Modules with more than 30 genes were obtained by dynamic pruning method. The correlation among different modules were calculated, and modules with a strong correlation can be merged into a same module. In addition, the module eigengenes were clustered according to *β* = 7, and the modules with dissimilarity less than 0.2 were selected for merging. According to the above screening criteria, a total of 26 modules were screened, among which the smallest module included 42 genes and the largest module included 2805 genes (Fig. 2a). The results of module eigengenes clustering and correlation among different modules of the 26 modules were shown in Fig. 2b.

### Screening and functional analysis of clinically key module

**Fig. 3:**
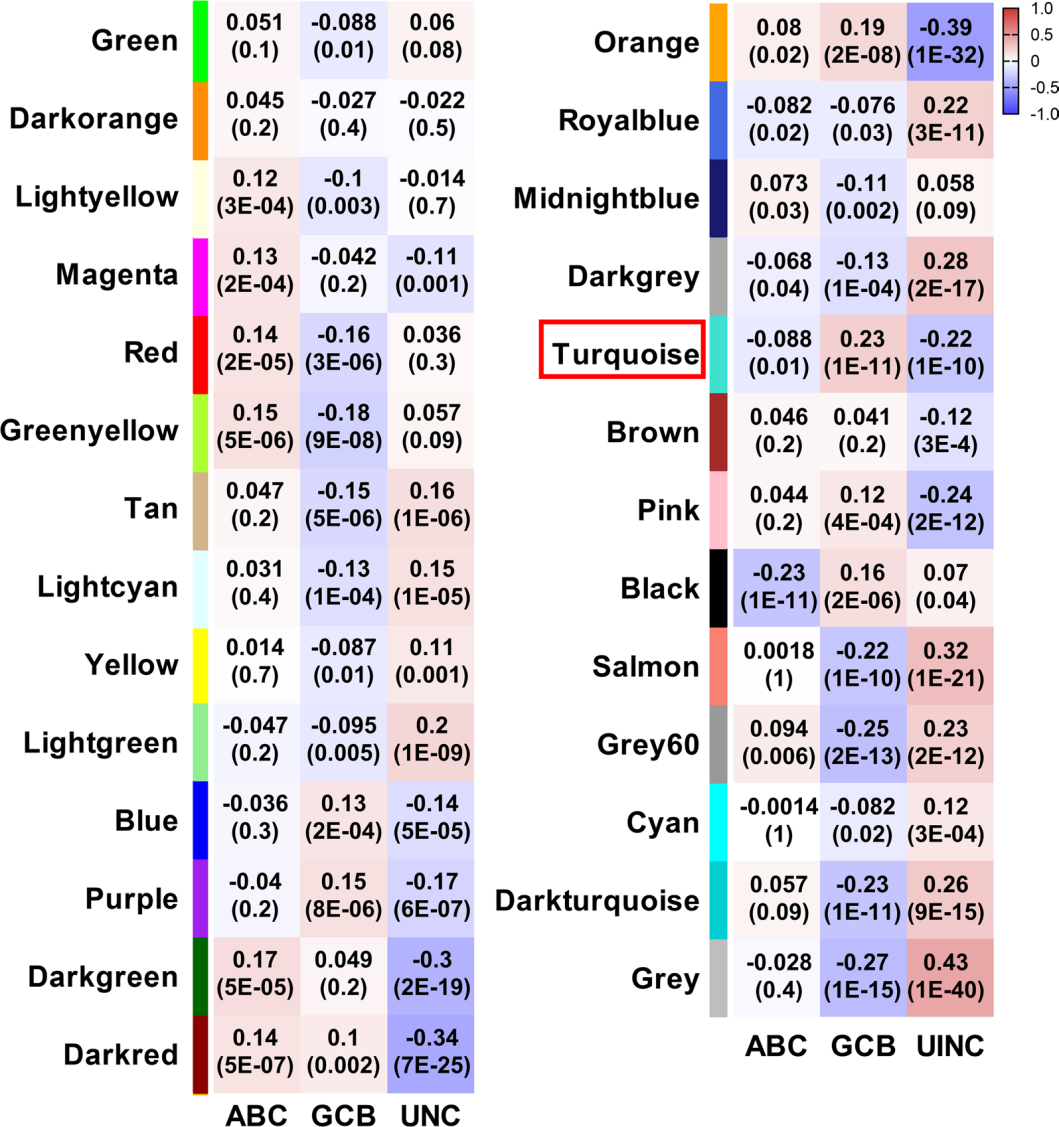
Identification of clinically key module of GCB-DLBCL. Correlation analysis between the 26 modules and different subtypes (ABC, GCB and UNC) of DLBCL. The numbers in each square represented correlation (r), and the numbers in parentheses of each square represented significance (*p*)

To obtain the clinically key modules, we calculated the correlation coefficient between the eigengenes value of the above 26 modules and the different pathological subtypes. As shown in Fig. 3, a total of 9 modules showed positive correlation with GCB subtype, among which turquoise module showed the highest correlation (r = 0.23) and the strongest significance (p = 1e-11). The turquoise module was a gene-set containing 2513 genes, among which the genes were strongly correlated.

**Fig. 4:**
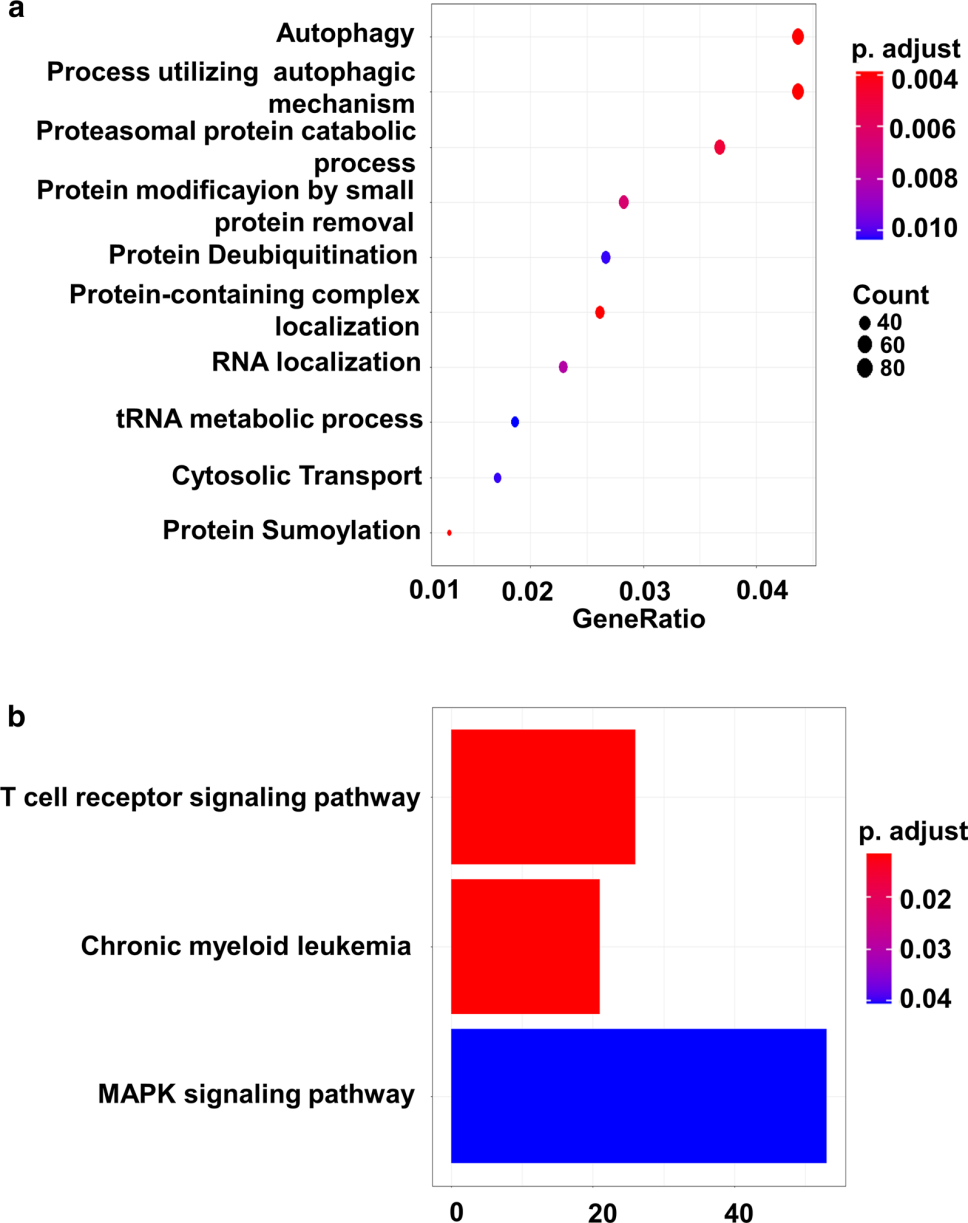
Functional analysis of genes in turquoise module. **a** The top 10 significant GO terms of genes in turquoise module. **b** KEGG analysis of genes in turquoise module. ME, module eigengene; GO, Gene ontology; KEGG, Kyoto Encyclopedia of Genes and Genomes

GO analysis of the genes in turquoise module found that the genes were mainly enriched in GO terms related to autophagy, protein modification and metabolism (Fig. 4a). KEGG analysis showed that the genes in turquoise module were enriched in “T cell receptor signaling pathway”, “chronic myeloid leukemia” and “MAPK signaling pathway” (Fig. 4b).

### Distribution of SEs in Karpas 422 and SUDHL-4 cells

**Fig. 5:**
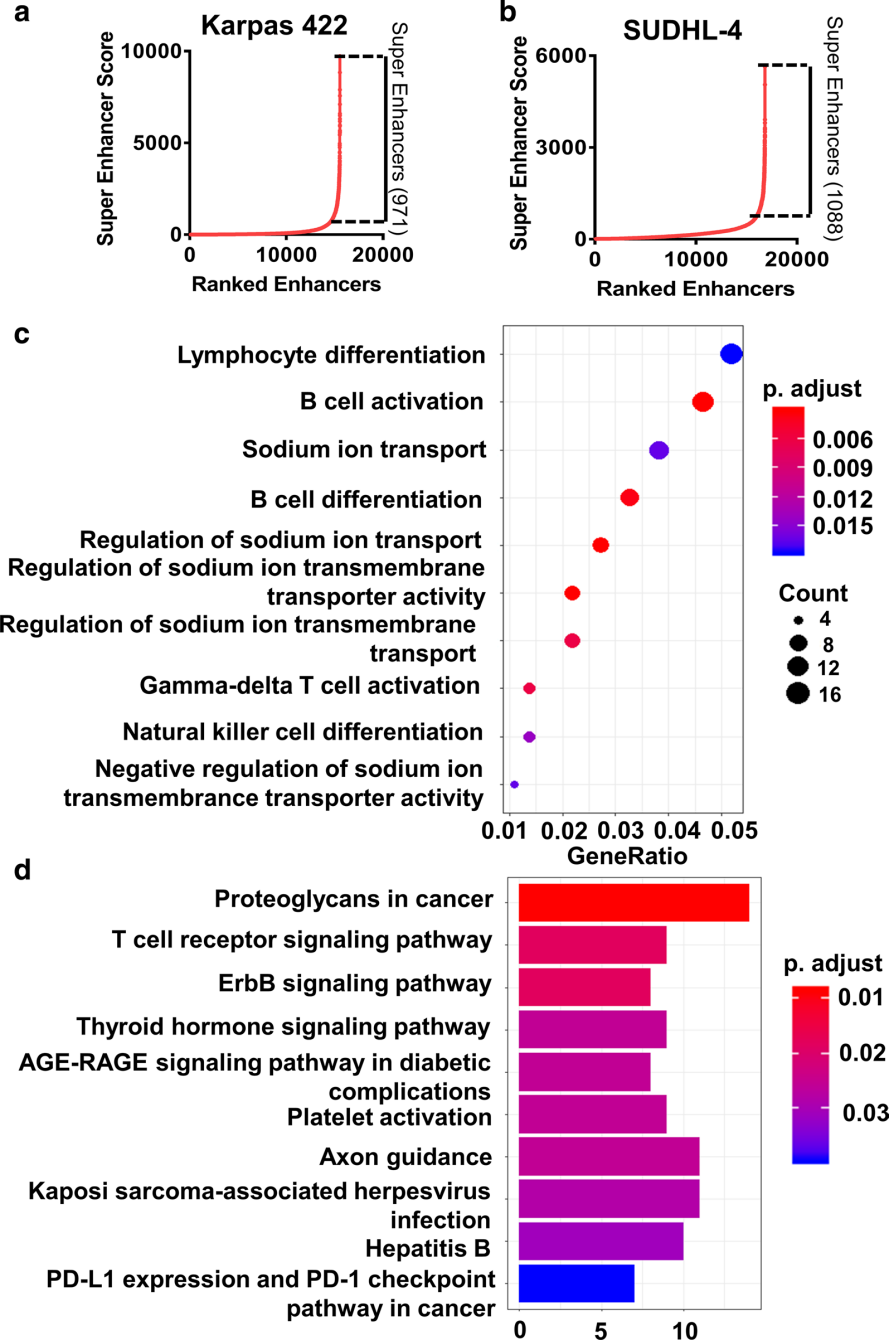
Identification of super enhancers in Karpas 422 and SUDHL-4 cells. Enhancers were ranked based on H3K27ac signal by HOMER algorithm. A total of 971 SE regions were identified in Karpas 422 cells **a**, and 1088 SE regions were screened in SUDHL-4 cells **b**. **c** The top 10 GO terms of the nearest genes of Karpas 422 and SUDHL-4 SEs. **d** The top 10 KEGG pathways of the nearest genes of Karpas 422 and SUDHL-4 SEs. GO, Gene ontology; KEGG, Kyoto Encyclopedia of Genes and Genomes

To investigate the regulation of SEs during GCB-DLBCL progression, we analyzed CHIP-Seq data of GCB-DLBCL cells (Karpas 422 and SUDHL-4 cells), and ranked enhancers according to the H3K27ac signal. A total of 15,544 enhancers were found in Karpas 422 cell, of which 971 enhancers were identified as SEs (Fig. 5a). In addition, 15,741 typical enhancers and 1088 SEs were characterized in SUDHL-4 cell (Fig. 5b).

GO annotation and KEGG pathway enrichment analysis were performed on the all of the nearest genes of Karpas 422 SEs and SUDHL-4 SEs. As shown in Fig. 5C, the top 5 GO terms of the nearest genes of SEs were “lymphocyte differentiation”, “B cell activation”, “sodium ion transport”, “B cell differentiation” and “regulation of sodium ion transport”. These annotated functions were mainly related to the differentiation and activation of lymphocytes. The top 10 KEGG pathways of the nearest genes of Karpas 422 SEs and SUDHL-4 SEs were presented in Fig. 5D. Many of these enrichment pathways, such as “proteoglycans in cancer”, “T cell receptor signaling pathway”, “ErbB signaling pathway” and “PD-L1 expression and PD-1 checkpoint pathway in cancer” were associated with cancer.

### Identification and prognostic analysis of SE-related genes associated with GCB-DLBCL

**Fig. 6:**
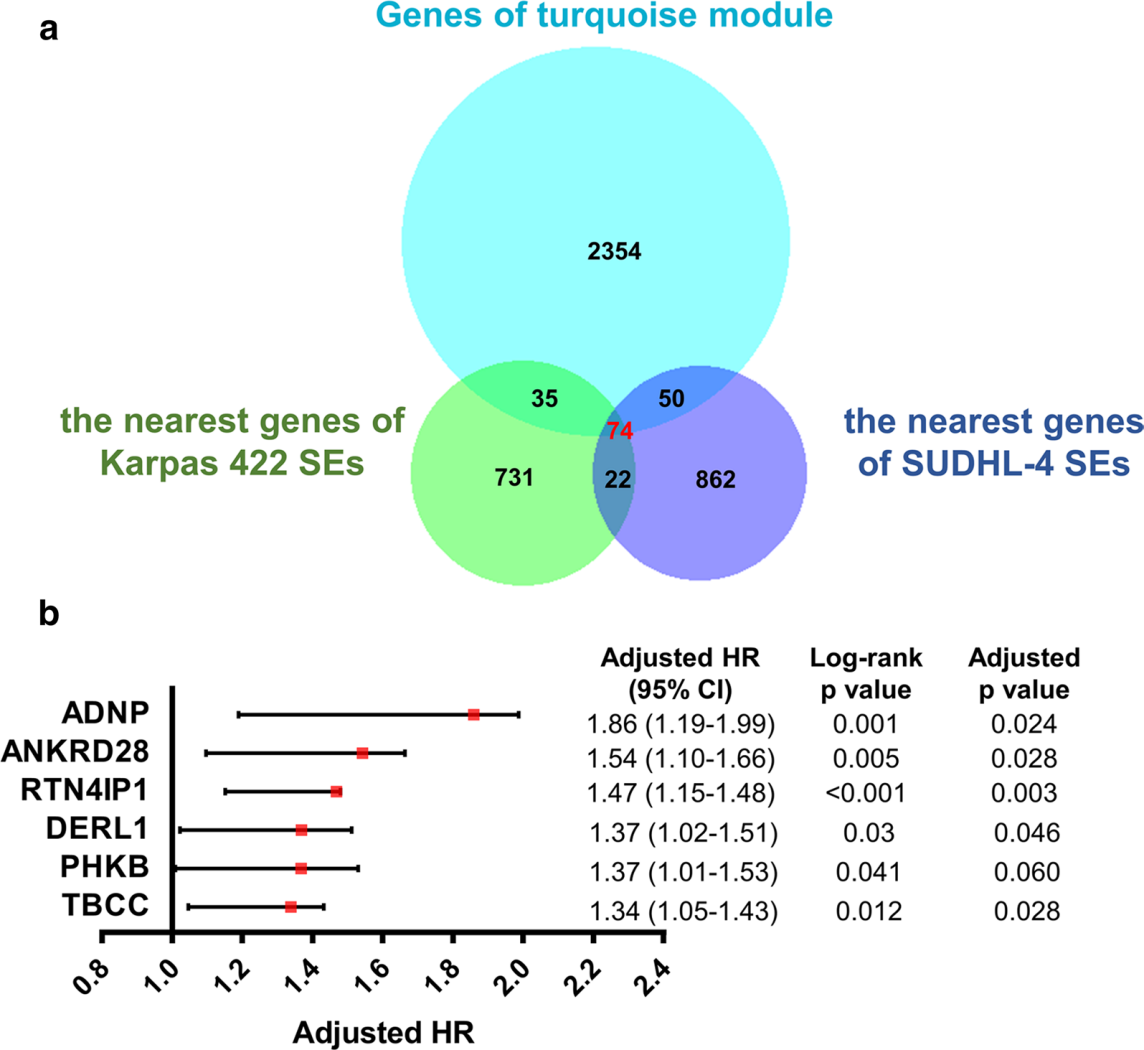
Identification and prognostic analysis of SE-related genes associated with GCB-DLBCL. **a** Venn plot of the nearest genes of Karpas 422 SEs (green), SUDHL-4 SEs (purple) and the genes of the turquoise module (blue). We annotated the gene nearest to the super enhancer as the SE-regulated gene, so each of the genes was located near a different super enhancer. **b** A total of 6 overlapping genes that significantly associated with the overall survival of GCB-DLBCL patients were screened using GSE117556 dataset. HR, hazard ratio; CI, confidence interval

Overlapping analysis was performed on the nearest genes of Karpas 422 SEs (n = 862), SUDHL-4 SEs (n = 1008) and the genes of the turquoise module (n = 2513) (Fig. 6a). A total of 74 overlapping genes were obtained (Fig. 6a). We annotated the gene nearest to the super enhancer as the SE-regulated gene, so each of the 74 genes was located near different super enhancers.

Kaplan–Meier survival analysis was applied to explore the overlapping gene expression on GCB-DLBCL progression based on GSE117556 dataset. A total of 6 of the 74 overlapping genes had significant associated (*p* < 0.05) with prognosis (Fig. 6b). Except for these 6 genes, the other overlapping genes had no significant association with the overall survival. The expression of ADNP, ANKRD28, RTN4IP1, DERL1, PHKB and TBCC significantly associated with increased risk of death, suggesting that the high expression of these genes contributes to GCB-DLBCL progression (Fig. 6b).

### The expression of ADNP, ANKRD28, RTN4IP1, DERL1, PHKB and TBCC in different pathological subtypes of DLBCL

**Fig. 7:**
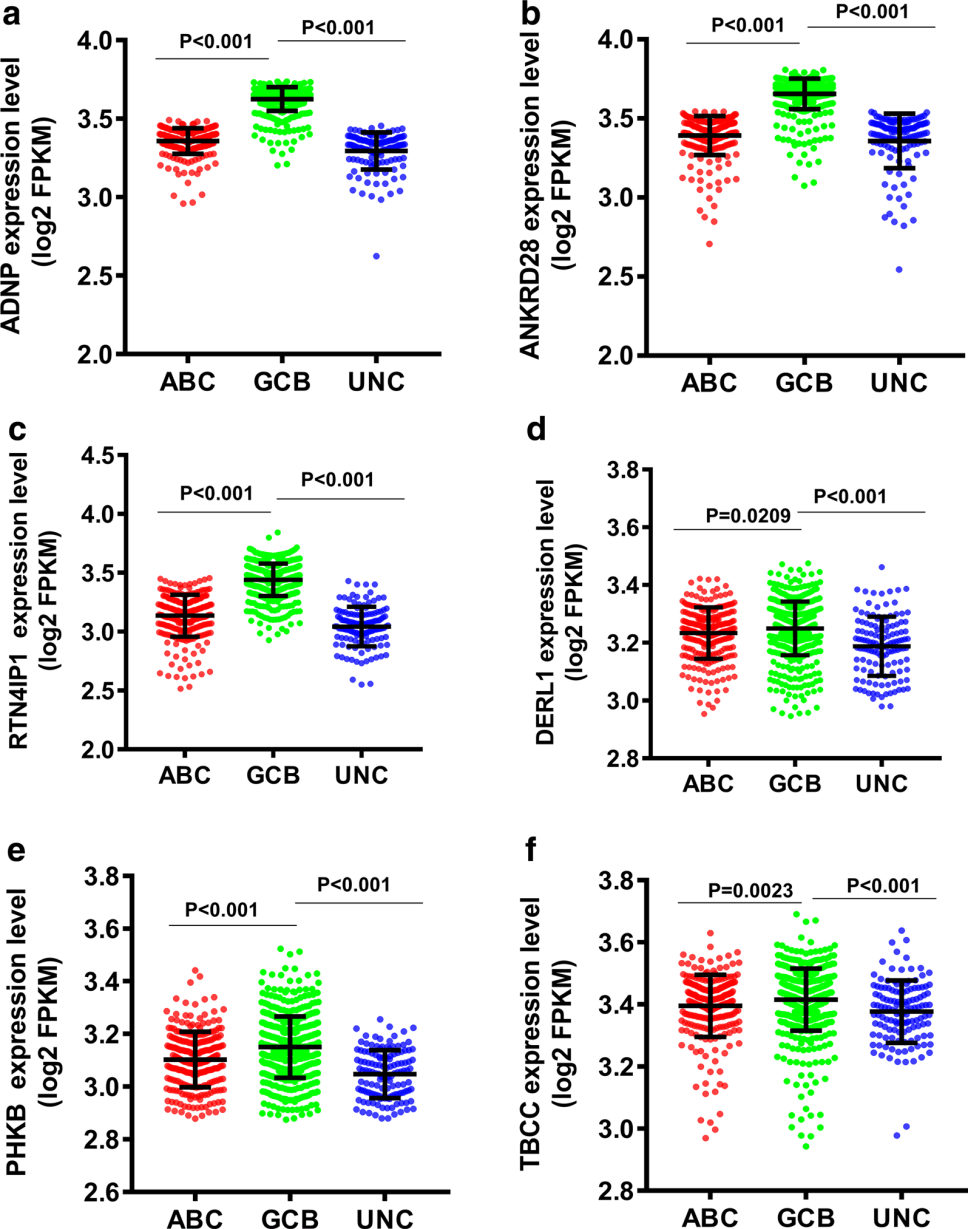
The expression of ADNP (**a**), ANKRD28 (**b**), RTN4IP1 (**c**), DERL1 (**d**), PHKB (**e**) and TBCC (**f**) in different pathological subtypes of DLBCL

Subsequently, we analyzed the expression of ADNP, ANKRD28, RTN4IP1, DERL1, PHKB and TBCC in ABC, GCB and UNC subtypes based on the GEO database. The expression levels of ADNP, ANKRD28, RTN4IP1, DERL1, PHKB and TBCC in GCB-DLBCL were significantly higher than those of ABC or UNC subtype (Fig. 7a–f).

### Identification of ADNP, ANKRD28 and RTN4IP1 in Karpas 422 and SUDHL-4 cells

**Fig. 8:**
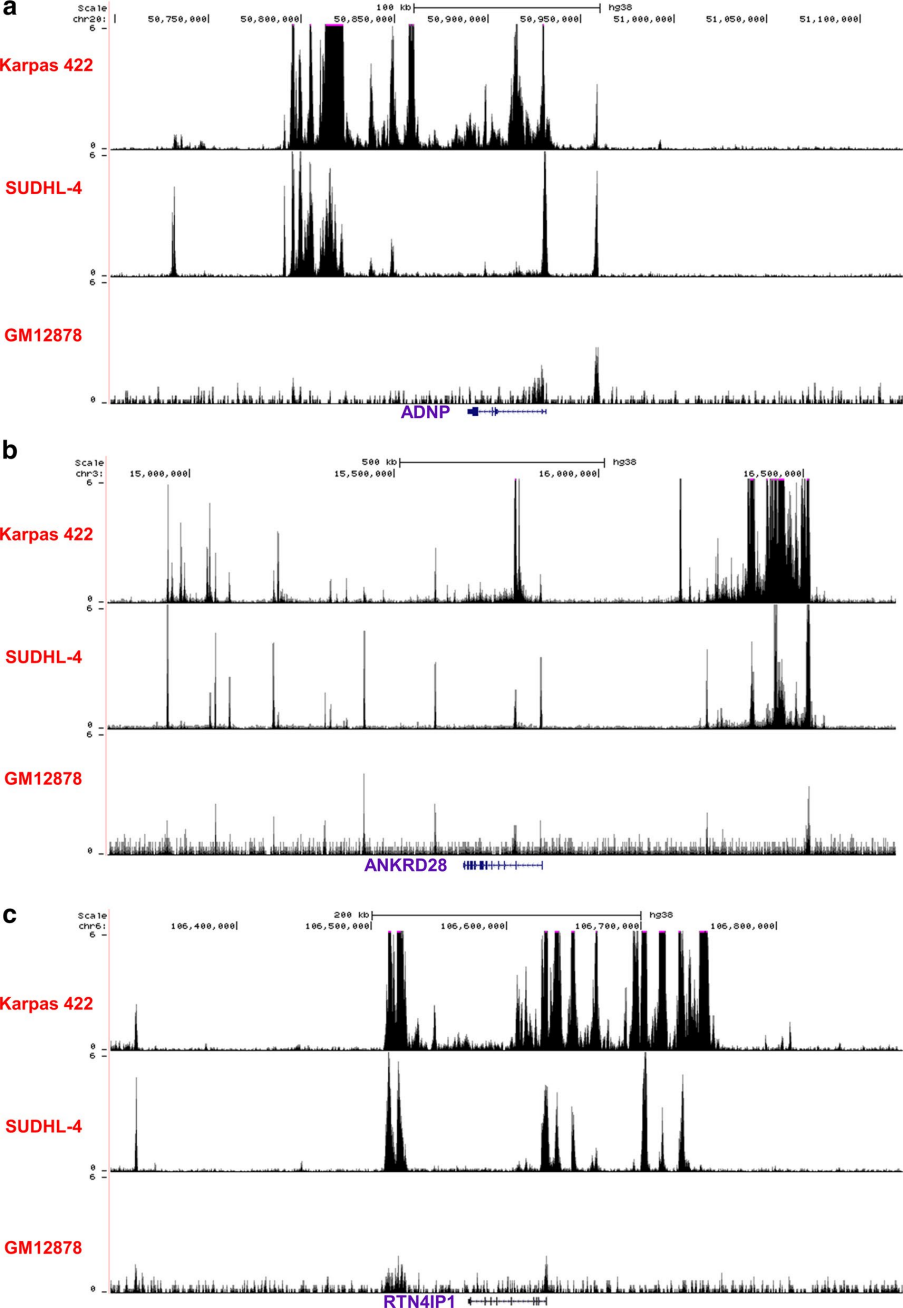
H3K27ac tracks at ADNP (**a**), ANKRD28 (**b**) and RTN4IP1 (**c**) locus in human lymphoblastoid B cells (GM12878) and GCB-DLBCL cells (Karpas 422 and SUDHL-4)

To further prove that ADNP, ANKRD28 and RTN4IP1 were regulated by SEs, the H3K27ac tracks of these gene locus in GCB-DLBCL cells were analyzed with a human lymphoblastoid B cell, GM12878, as control. As illustrated in Fig. 8, Karpas 422 and SUDHL-4 cells had SEs at ADNP, ANKRD28 and RTN4IP1 locus. However, there was no super enhancer at ADNP, ANKRD28 and RTN4IP1 locus in GM12878 cell (Fig. 8). These results suggested that ADNP, ANKRD28 and RTN4IP1 in GCB-DLBCL cell were regulated by SEs, and these SEs were cancer-associated.

**Fig. 9:**
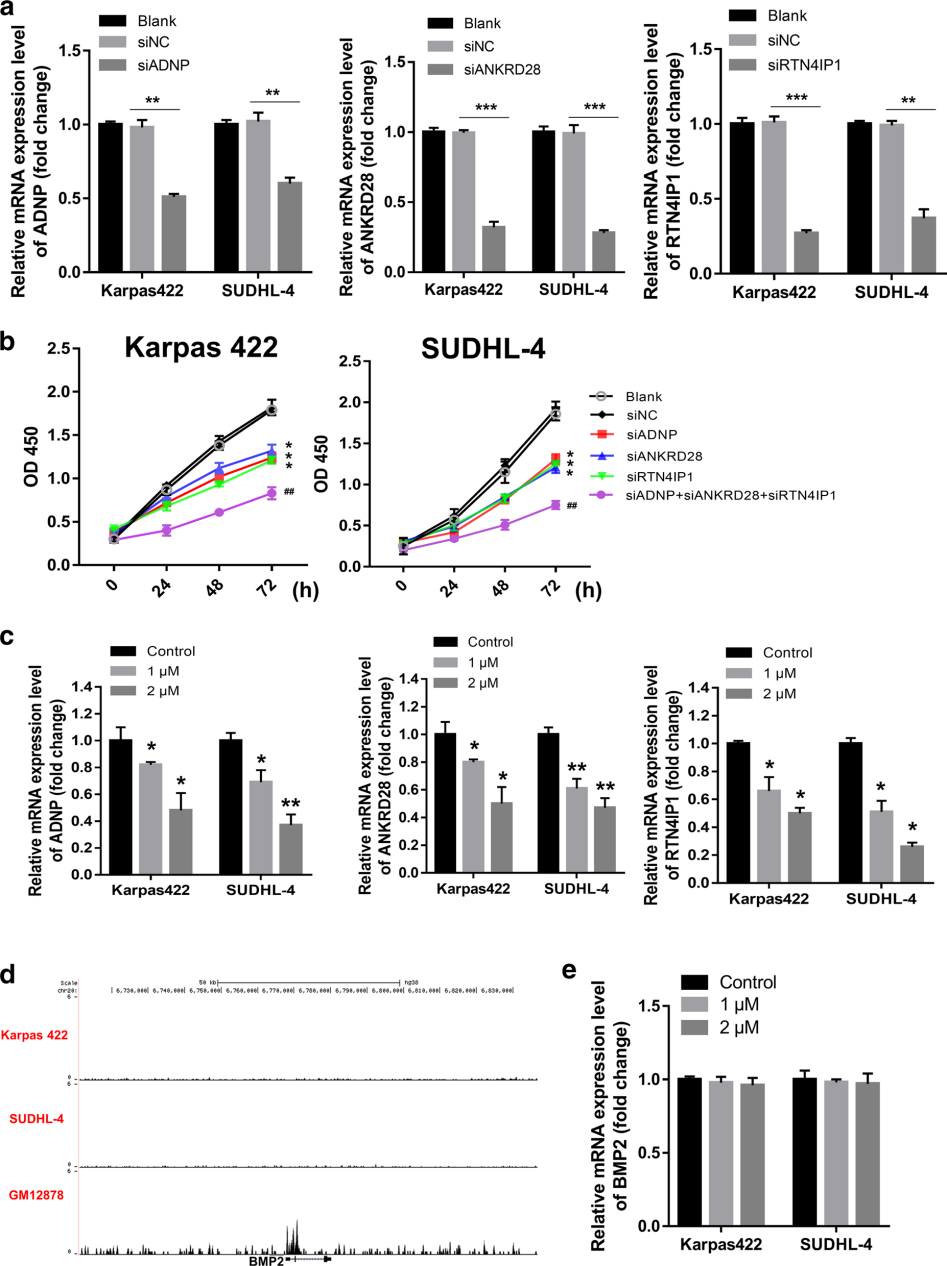
Identification of ADNP, ANKRD28 and RTN4IP1 in Karpas 422 and SUDHL-4 cells. **a** qRT-PCR was performed to validate the knockdown efficiency of siADNP, siANKRD28 and siRTN4IP1 in Karpas 422 and SUDHL-4 cells. **p < 0.01; ***p < 0.001. **b** The effect of ADNP, ANKRD28 or RTN4IP1 knockdown on cell proliferation were detected using CCK-8 method. *p < 0.05, siADNP, siANKRD28 or siRTN4IP1 group vs. siNC group. ^##^*p* < 0.01, siADNP + siANKRD28 + siRTN4IP1 group vs. siADNP, siANKRD28 and siRTN4IP1 group. **c** The relative mRNA expression levels of ADNP, ANKRD28 and RTN4IP1 in Karpas 422 and SUDHL-4 cells treated with 0, 1 or 2 μM JQ1. *p < 0.05, 1 or 2 μM JQ1 groups vs. control group. **p < 0.01, 1 or 2 μM JQ1 groups vs. control group. **d** A gene, BMP2, without SE in Karpas 422 and SUDHL-4 cells were screened out. **e** The relative mRNA expression levels of BMP2 in Karpas 422 and SUDHL-4 cells treated with 0, 1 or 2 μM JQ1

The top 3 key SE-associated genes (ADNP, ANKRD28 and RTN4IP1) with the most robust association with prognosis were selected for cellular level verification. ADNP, ANKRD28 and RTN4IP1 were successfully knocked down in Karpas 422 and SUDHL-4 cells (Fig. 9a). Knockdown of ADNP, ANKRD28 or RTN4IP1 significantly inhibited the proliferation of Karpas 422 and SUDHL-4 cells (Fig. 9b). In addition, co-transfection of siADNP, siANKRD28 and siRTN4IP1 further reduced cell proliferation compared with knockdown of ADNP, ANKRD28 or RTN4IP1 separately (Fig. 9b).

Finally, Karpas 422 and SUDHL-4 cells were treated with different concentrations of the bromodomain and extra-terminal domain (BET) inhibitor JQ1 [16, 18]. The effects of JQ1 treatment on ADNP, ANKRD28 and RTN4IP1 expression were measured by qRT-PCR. JQ1 treatment significantly decreased ADNP, ANKRD28 and RTN4IP1 expression in Karpas 422 and SUDHL-4 cells (Fig. 9c). Furthermore, we screened a gene, BMP2, without SE in Karpas 422 and SUDHL-4 cells (Fig. 9d). As expected, JQ1 treatment had no significant effect on the expression of BMP2 in Karpas 422 and SUDHL-4 cells (Fig. 9e).

## Discussion

Genetic abnormalities are important initiation factors for the development of GCB-DLBCL, the role of epigenetic changes in the disease process has received increasing attention. In this study, we identified cancer-associated super enhancers in GCB-DLBCL cells. These super enhancers were significantly enriched in tumor-related biological processes and signaling pathways.

Studies have shown that there are usually some genes with common expressions in disease samples, and their co-expression is closely related to disease phenotype. Gene co-expression network analysis is an important method to discover these genes [19]. In this study, 26 modules of DLBCL were identified using WGCNA, among which the turquoise module was closely related to the GCB subtype. DLBCL is a highly heterogeneous tumor. It should be noted that although previous studies have divided DLBCL into three types: ABC, GCB and UNC, there is still strong heterogeneity in GCB subtype [20]. Although the correlation between the turquoise module and GCB seems to be “weak”, the data set selected in this study has a larger number of samples (including 255 ABC, 543 GCB and 130 UNC), and the correlation between turquoise module and GCB was statistically significant. Therefore, we believed that the turquoise module still had great research significance. Functional analysis showed that the function of genes in turquoise module was mainly related to autophagy. Autophagy is a classical regulatory mechanism that maintains homeostasis and cell development [21]. Autophagy has been recently found to play key roles in tumor development, proliferation, metastasis and metabolism [22]. Studies have shown that malignant transformation of mature B cells requires mutations that impair the intrinsic differentiation process and allow for evasion of T cell-mediated tumor monitoring [23, 24]. Consistent with previous studies, other biological pathways, including “T cell receptor signaling pathway” and “MAPK signaling pathway”, were also highly altered [25, 26]. These results indicated that the aberrant expression of genes in turquoise module might lead to the disruption of core cancer-signaling pathways, and contribute to carcinogenesis and progression of GCB-DLBCL.

SE is a large cluster of transcriptionally active enhancers that drive the expression of cellular identity genes and play critical roles in the development of diseases such as tumors [15]. Many tumor cell key oncogenes are driven by SEs compared to normal enhancers [16]. SEs show great potential in key oncogenes identification and disease-associated variant sites discovery [27]. In this study, we identified 971 SEs in Karpas 422 cell, and 1088 SEs in SUDHL-4 cell. The function of all of the nearest genes of Karpas 422 SEs and SUDHL-4 SEs were principally associated with lymphocytes and cancer. Moreover, 74 SE-related genes associated with GCB-DLBCL were further subjected to overall survival analysis, and 6 genes (ADNP, ANKRD28, RTN4IP1, DERL1, PHKB and TBCC) were identified as prognostic markers. In particular, the 6 genes were specifically expressed in GCB-subtype. Further experimental verification showed that knockdown of ADNP, ANKRD28 and RTN4IP1 markedly inhibited the proliferation of Karpas 422 and SUDHL-4 cells. SEs can be specifically recognized by the BET, thereby recruiting chromatin regulatory factors to specific regions to coordinate gene expression regulation [16, 18]. Following treatment with the BET inhibitor, JQ1, the expression levels of ADNP, ANKRD28 and RTN4IP1 were reduced. These findings implied that ADNP, ANKRD28 and RTN4IP1 were regulated by SEs and significantly related to GCB-DLBCL progress. Among them, ADNP is usually up-regulated in most cancers such as ovarian cancer and colorectal cancer [28, 29]. In addition, ANKRD28 has been confirmed as an oncogene in acute myeloid leukemia [30, 31]. However, the regulation of ADNP and ANKRD28 on the SEs in DLBCL remains to be further studied. Other genes, such as RTN4IP1 and TBCC, have been reported to be related to breast cancer progression [^32, 33^]. DLBCL is a group of tumors with biological heterogeneity, and clinical prognosis of different subtypes varies greatly. DLBCL can be divided into three groups: GCB, ABC and UNC according to gene expression. However, there is no "necessary connection" between the molecular typing of DLBCL and treatment options. The overexpression of SE-regulated genes provides the possibility for SEs and their regulated genes as markers in tumor diagnosis and treatment. These candidate prognostic genes will be useful in improving prognosis prediction accuracy in combination with other genetic and transcriptional events of DLBCL.

## Conclusions

In summary, we identified SEs in GCB-DLBCL by WGCNA and enhancer signatures. These results revealed that altered SE patterns were involved in key cancer-signaling pathways known to be important in GCB-DLBCL tumorigenesis. In addition, 6 SE-related genes were identified as candidate markers for GCB-DLBCL prognosis. These results will provide a basis for further study on the mechanism of DLBCL progress.

## Supplementary Information

**Additional file 1: Table S1.** Primer sequences of qRT-PCR.

## Data Availability

The data set in this study can be obtained under reasonable conditions by contacting the corresponding author (Email: yuxiahao_678@163.com). GSE117556 and GSE69558 datasets were obtained from Gene Expression Omnibus (GEO) (https://www.ncbi.nlm.nih.gov/geo/) database. ENCSR660IQS dataset was obtained from Encyclopedia of DNA Elements (ENCODE) (https://www.encodeproject.org/).

## Abbreviations

GCB: Germinal center B-cell type
DLBCL: Diffuse large B-cell lymphoma
SEs: Super enhancers
WGCNA: Weighted gene co-expression network analysis
GO: Gene ontology
KEGG: Kyoto encyclopedia of genes and genomes
BET: Bromodomain and extra-terminal domain
ABC: Activated B-cell type
UNC: Unclassified type
GEO: Gene expression omnibus
TOM: Topological overlap matrix
ME: Module eigengene
CI: Confidence interval
HRs: Hazard ratios
ATCC: American type culture collection
CCK-8: Cell counting kit-8
