# Supplementary material for: Identification of super enhancer-associated key genes for prognosis of germinal center B-cell type diffuse large B-cell lymphoma by integrated analysis
Source: BMC Med Genomics. 2021 Mar 4;14:69. doi: 10.1186/s12920-021-00916-z (PMC7934469; doi:10.1186/s12920-021-00916-z)
Supplement: Supplementary file 1 — Additional file 1: Table S1. Primer sequences of qRT-PCR. [file 12920_2021_916_MOESM1_ESM.docx]

| Gene | Primer sequences | |
| --- | --- | --- |
| ADNP | forward | 5’-CATGGGAGGATGTAGGACTGT-3’ |
|  | reverse | 5’-ATGGACATTGCGGAAATGACT-3’ |
| ANKRD28 | forward | 5’- AATTGCTTGTGTCGCATGGAG-3’ |
|  | reverse | 5’-TAGCAGGCTACATGAAGAGGT-3' |
| RTN4IP1 | forward | 5’- TGCCTGCTTGGGTGATAGATA-3’ |
|  | reverse | 5’- TGCCTGCTTGGGTGATAGATA-3’ |
| GAPDH | forward | 5’-TGTGGGCATCAATGGATTTGG-3’ |
|  | reverse | 5’-ACACCATGTATTCCGGGTCAAT-3’ |

**Table S1** Primer sequences of qRT-PCR.
